# Response to SGLT2 inhibitors in heart failure with tricuspid regurgitation: clinical interpretation and future directions

**DOI:** 10.1093/ehjcvp/pvag022

**Published:** 2026-04-11

**Authors:** Ranel Loutati, Viana Copeland, Shir Elimeleh, David Hochstein, Kobi Faierstein, Assi Milwidsky, Sagit Ben-Zekry, Amit Segev, Rafael Kuperstein, Elad Maor

**Affiliations:** The Olga and Lev Leviev Heart Center, Chaim Sheba Medical Center Hospital, 2 Derech Sheba, Tel Hashomer, Ramat Gan 52621, Israel; School of Medicine, Tel Aviv University, Ramat Aviv, Tel Aviv 6997801, Israel; The Olga and Lev Leviev Heart Center, Chaim Sheba Medical Center Hospital, 2 Derech Sheba, Tel Hashomer, Ramat Gan 52621, Israel; School of Medicine, Tel Aviv University, Ramat Aviv, Tel Aviv 6997801, Israel; The Olga and Lev Leviev Heart Center, Chaim Sheba Medical Center Hospital, 2 Derech Sheba, Tel Hashomer, Ramat Gan 52621, Israel; School of Medicine, Tel Aviv University, Ramat Aviv, Tel Aviv 6997801, Israel; The Olga and Lev Leviev Heart Center, Chaim Sheba Medical Center Hospital, 2 Derech Sheba, Tel Hashomer, Ramat Gan 52621, Israel; School of Medicine, Tel Aviv University, Ramat Aviv, Tel Aviv 6997801, Israel; The Olga and Lev Leviev Heart Center, Chaim Sheba Medical Center Hospital, 2 Derech Sheba, Tel Hashomer, Ramat Gan 52621, Israel; School of Medicine, Tel Aviv University, Ramat Aviv, Tel Aviv 6997801, Israel; The Olga and Lev Leviev Heart Center, Chaim Sheba Medical Center Hospital, 2 Derech Sheba, Tel Hashomer, Ramat Gan 52621, Israel; School of Medicine, Tel Aviv University, Ramat Aviv, Tel Aviv 6997801, Israel; The Olga and Lev Leviev Heart Center, Chaim Sheba Medical Center Hospital, 2 Derech Sheba, Tel Hashomer, Ramat Gan 52621, Israel; School of Medicine, Tel Aviv University, Ramat Aviv, Tel Aviv 6997801, Israel; The Olga and Lev Leviev Heart Center, Chaim Sheba Medical Center Hospital, 2 Derech Sheba, Tel Hashomer, Ramat Gan 52621, Israel; School of Medicine, Tel Aviv University, Ramat Aviv, Tel Aviv 6997801, Israel; The Olga and Lev Leviev Heart Center, Chaim Sheba Medical Center Hospital, 2 Derech Sheba, Tel Hashomer, Ramat Gan 52621, Israel; School of Medicine, Tel Aviv University, Ramat Aviv, Tel Aviv 6997801, Israel; The Olga and Lev Leviev Heart Center, Chaim Sheba Medical Center Hospital, 2 Derech Sheba, Tel Hashomer, Ramat Gan 52621, Israel; School of Medicine, Tel Aviv University, Ramat Aviv, Tel Aviv 6997801, Israel

**This correspondence refers to ‘SGLT2 inhibitors in heart failure with tricuspid regurgitation: clinical interpretation and future directions’, by W. Dong *et al*., https://doi.org/10.1093/ehjcvp/pvag021.**

We thank Dong *et al*. for their thoughtful engagement with our work and for the opportunity to further clarify several methodological and interpretative aspects of our study.^1^

First, we agree that the timing of sodium-glucose cotransporter-2 inhibitors (SGLT2i) initiation in heart failure (HF) is clinically meaningful.^2^ As our analysis relied on a large real-world registry, SGLT2i use was evaluated irrespective of initiation timing, and treatment exposure was modelled using time-dependent methods. While this approach allows us to better approximate real-world prescribing patterns, it does not permit detailed evaluation of how treatment timing influences outcomes, an important limitation that we acknowledge. Future prospective studies with structured follow-up and serial echocardiographic assessments will be essential to determine chamber-specific remodelling and tricuspid regurgitation (TR) dynamics following initiation of SGLT2i therapy.

Second, the hypothesis raised regarding the potential link among SGLT2i therapy, atrial fibrillation (AF), and attenuation of TR progression is compelling. Given the established relationship between atrial arrhythmias, right atrial remodelling, and TR severity,^3^ rhythm-related outcomes may indeed offer mechanistic insights. As Dong et al. note, emerging evidence suggests an association between SGLT2i therapy and reduced AF incidence.^4^ We agree that mechanistic work integrating echocardiography with electrophysiologic evaluation would be valuable as briefly discussed in our manuscript.

Third, we appreciate the suggestion to explore the broader implications of SGLT2i use across cardiovascular populations, including patients with TR irrespective of HF status. Although current indications for SGLT2i therapy remain limited to type 2 diabetes, chronic kidney disease, and HF, expanding evidence suggests potential benefit across a wider cardiovascular spectrum. Our focus on HF patients reflects both the present therapeutic landscape and the disproportionately high burden of clinically relevant TR in this population. We share the authors’ anticipation that future studies, including those evaluating valvular disease subgroups, will help clarify the therapeutic reach of SGLT2i in cardiovascular medicine.

Fourth, we agree that real-world eligibility and implementation gaps represent an important dimension of SGLT2i research.^5^ Trial-based inclusion criteria often diverge from clinical practice, and prescription patterns remain suboptimal worldwide. We did not explicitly examine eligibility alignment or prescribing variability in our study, but these factors undoubtedly influence treatment uptake and outcome associations. Incorporating these considerations into future work would strengthen interpretation and highlight targets for improving guideline-directed care.

Finally, we concur that optimization of medical therapy is essential prior to proceeding with transcatheter or surgical interventions for TR. Our study provides observational evidence supporting the potential role of SGLT2i therapy in HF patients with TR, but interventional strategies should always be considered in the context of optimized guideline-directed therapy. We hope that our findings will stimulate clinical and translational studies assessing how pharmacologic and interventional approaches can be integrated to improve outcomes in this high-risk population.

In summary, we appreciate the insightful comments by Dong et al. Our study offers preliminary real-world evidence suggesting favourable associations of SGLT2i therapy in HF patients with concomitant TR. We believe these findings lay the groundwork for future mechanistic, implementation, and interventional studies aimed at improving the care of patients with HF and TR.

## Data Availability

No new data were generated or analysed in support of this manuscript.
